# Fish bone foreign body presenting with an acute fulminating retropharyngeal abscess in a resource-challenged center: a case report

**DOI:** 10.1186/1752-1947-5-165

**Published:** 2011-04-27

**Authors:** Olushola A Afolabi, Joseph O Fadare, Ezekiel O Oyewole, Stephen A Ogah

**Affiliations:** 1 Kogi State Specialist Hospital, Lokoja, Kogi State, Nigeria; 2 University of Ilorin Teaching Hospital, Ilorin, Kwara State, Nigeria

## Abstract

**Introduction:**

A retropharyngeal abscess is a potentially life-threatening infection in the deep space of the neck, which can compromise the airway. Its management requires highly specialized care, including surgery and intensive care, to reduce mortality. This is the first case of a gas-forming abscess reported from this region, but not the first such report in the literature.

**Case presentation:**

We present a case of a 16-month-old Yoruba baby girl with a gas-forming retropharyngeal abscess secondary to fish bone foreign body with laryngeal spasm that was managed in the recovery room. We highlight specific problems encountered in the management of this case in a resource-challenged center such as ours.

**Conclusion:**

We describe an unusual presentation of a gas-forming organism causing a retropharyngeal abscess in a child. The patient's condition was treated despite the challenges of inadequate resources for its management. We recommend early recognition through adequate evaluation of any oropharyngeal injuries or infection and early referral to the specialist with prompt surgical intervention.

## Introduction

A retropharyngeal abscess is an infection with abscess collection in one of the deep spaces of the neck [1-3]. An abscess in this location is an immediate life-threatening emergency with the potential for airway compromise and other catastrophic complications [1]. Patients with diabetes and those who are debilitated, older adults or immunocompromised patients are more likely to get this infection [2-4]. Delay in diagnosis results in high mortality and morbidity [4,5]. Although much has been written about this clinical condition and its clinical indicators, this case report is the first case of a gas-forming retropharyngeal abscess in a child with a foreign body (a fish bone) seen in North-central part of Nigeria. This particular case was challenging as the child developed a laryngeal spasm postoperatively but was managed in the recovery room without a stay in the intensive care unit (ICU). Other challenges were inadequate laboratory facilities. Laryngospasm is a forceful, involuntary spasm of the laryngeal musculature, and its symptoms include inability to help the patient ventilate with resultant rapid desaturation, which requires ICU care. We emphasize early recognition, prevention of oropharyngeal trauma and prompt surgical intervention for life-threatening head and neck infections, even in the face of challenges.

## Case presentation

A 16-month-old Yoruba girl was referred from a peripheral hospital to the ear, nose and throat (ENT) unit of our hospital with a one-week history of fever, a six-day history of cough and a five-day history of neck swelling.

Her fever was high grade with bouts of cough, and she had no history of contact with a person with chronic cough, no associated weight loss and no posttussive vomiting. Her mother noticed neck swelling five days before presentation which was progressive and painful, with associated limited neck movement. The patient refused to eat, expectorated a thick tenacious secretion, and had episodes of irritability and excessive crying. The child had a previous history of left ear discharge which had resolved, and there was no history of hearing impairment or nasal symptoms. About three days prior to presentation, the child was noticed to be breathless, for which she was treated at a private hospital as a case of pneumonia and was placed on an antitussive and antibiotics.

The patient's medical history and family and social history, as well as the review of systems, were not remarkable. An examination of the throat revealed poor oral hygiene; foul-smelling, thick, tenacious, straw-colored secretion from the oral cavity and oropharynx; and a bulging posterior pharyngeal wall. The patient's neck showed a diffuse swelling which was tender. The ear, nose, chest and abdominal examinations were essentially normal.

An assessment of retropharyngeal abscess was made to rule out parapharyngeal abscess. Investigations revealed that the packed cell volume was 41%, and the electrolyte and urea examinations showed the following concentrations: sodium, 142 mM/L; potassium, 3.7 mM/L; urea 6.5 mM/L; and creatinine, 101 mM/L.

**Figure 1:**
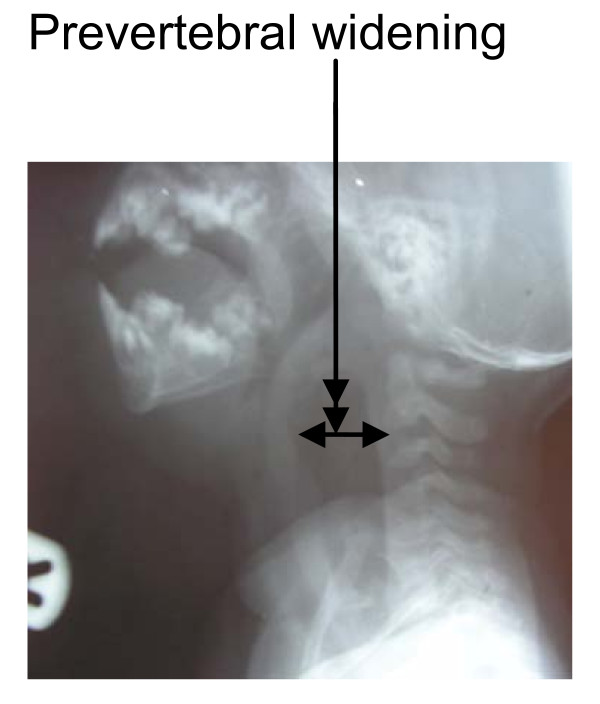
**Lateral view X-ray showing the soft neck tissue and revealing widening of the prevertebral space containing areas of mixed opacity and lucency extending from the base of the skull to the level of the seventh cervical spine (C7), with the laryngeal air column almost obliterated, anterior displacement of the airway and straightening of the cervical spine**.

**Figure 2:**
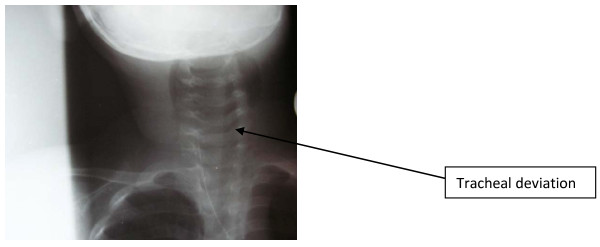
**Anteroposterior view X-ray showing lateral displacement of the trachea to the left**.

X-rays of the soft neck tissue revealed widening of the prevertebral space containing areas of opacity and lucency extending from the base of the skull to the level of the seventh cervical spine (C7), which at the level of the second cervical vertebra (C2) was about 22 mm, with the laryngeal air column almost obliterated and anterior displacement of the airway and straightening of the cervical spine (Figure 1). There was lateral displacement of the trachea to the left from the anteroposterior view (Figure 2).

The patient was resuscitated with intravenous fluid and antibiotics and was taken for examination under anesthesia and drainage of the abscess. The patient was placed in the anti-Trendelenburg position while under general anesthesia. Intubation was difficult but was finally achieved using a size 2.5 mm endotracheal tube inserted by an experienced anesthetist, and light packing with wet gauze was placed around the endotracheal tube. Anesthesia was induced with halothane in oxygen, and the trachea was secured with 1 mg/kg suxamethonium. Anesthesia was maintained with 66% nitrous oxide in oxygen and 0.5% to 1% halothane in oxygen, while muscle paralysis was induced with 0.1 mg/kg pancuronium. Analgesia was ensured with 2 μg/kg fentanyl.

**Figure 3:**
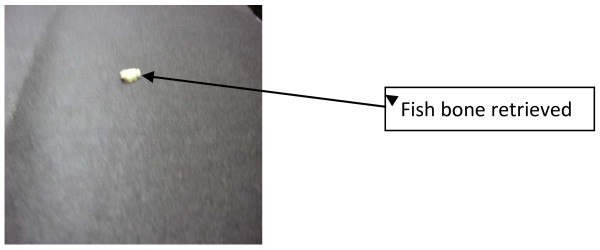
**Photograph of fish bone (foreign body) remnant removed from the abscess cavity**.

A Boyle-Davis mouth gag was introduced gently to expose the oral cavity and oropharynx, a cruciate incision was made using a size 11 surgical blade and a surgical probe was introduced to break down all loculi. About 30 to 40 mL of foul-smelling, purulent discharge was drained with the extrusion of a fish bone remnant from the abscess cavity (Figure 3). The culture revealed a growth of mixed organisms: *Staphylococcus aureus*, *Klebsiella pneumoniae *and anaerobic streptococci. Prior to extubation, residual neuromuscular block was antagonized with a combination of 0.04 mg/kg neostigmine and 0.02 mg/kg atropine. The patient was extubated but suddenly developed laryngeal spasm. Manual ventilation with a face mask was difficult as the patient's pulse oximetry was less than 80%. Anesthesia was deepened with halothane, and the patient's trachea was resecured with 1 mg/kg suxamethonium. The patient was ventilated manually with 100% oxygen in the improvised recovery room on account of poor respiratory function for about 8 to 10 hours, after which she was transferred to the postoperative ward, where her condition was satisfactory. The patient was maintained on intravenous antibiotics, analgesics and anti-inflammatory agents. The patient was discharged to home on the fifth day postoperatively.

## Discussion

Retropharyngeal abscess is not common nowadays with the increasing use of antibiotics in the treatment of upper respiratory tract infections. It is almost exclusively a pediatric diagnosis. Most incidents occur in children ages six months to six years [3-6] in whom the index case still falls within a mean age of three to four years [2-4]. No racial or sex predilection has been described in the literature, but several studies have noted a higher incidence of deep neck space infections in boys [3,4], which is at variance with our present report of the case of a young girl.

The retropharyngeal space is located immediately posterior to the nasopharynx, oropharynx, hypopharynx, larynx and trachea [3,5]. The visceral (that is, buccopharyngeal) fascia, which surrounds the pharynx, trachea, esophagus and thyroid, forms the anterior border of the retropharyngeal space. Bounded posteriorly by the alar fascia, the retropharyngeal space is bounded laterally by the carotid sheaths and parapharyngeal spaces [5]. It extends superiorly to the base of the skull and inferiorly to the mediastinum at the level of the tracheal bifurcation.

The retropharyngeal space can become infected in three ways [3,4]. Either infection spreads from a contiguous area affecting the retropharyngeal nodes or the space is inoculated directly secondary to a penetrating foreign body as we observed in our case in which a fish bone foreign body penetrated the retropharyngeal space as found intraoperatively. It may be through oropharyngeal injuries such as accidental lacerations, which are not uncommon in children who run and fall down after they have placed an object such as a toy, stick, pencil or toothbrush into their mouths [4,7-10]. There also are iatrogenic causes, which include instrumentation with laryngoscopy, endotracheal intubation, surgery, endoscopy, feeding tube placement and dental injection procedures [11], which inoculate these organisms directly into the retropharyngeal space.

Our index case was initially managed for pneumonia by the general practitioner; however, there is a need to encourage caregivers to present their children for treatment early. The diagnosis of this condition is mainly clinical, with some support from the radiological investigation, which can also occasionally be confirmatory. Patients with retropharyngeal abscess may present with airway compromise, thus the management of the airway takes priority with regard to patient care. Fortunately, our patient did not present with airway challenges, except postoperatively. The culture in the present case revealed mixed aerobic and anaerobic flora with gas-forming organisms (that is, *Klebsiella*, anaerobic streptococci). The other gas-forming organisms isolated from the head and neck infections described in previous reports are *Clostridium *[12], *Bacteroides *and *Fusobacterium *[13].

Patients with retropharyngeal abscess present with constitutional complaints such as fever, chills, malaise, decreased appetite, muffled "hot potato" voice [4] and irritability [2] as seen in our index case. Older patients may complain of sore throat, dysphagia, odynophagia, trismus or torticollis; however, our index case was an infant who was unable to demonstrate the expected symptoms, although she refused to eat [1,3,4,9].

A lateral soft tissue neck X-ray is contributory in making the diagnosis of a retropharyngeal abscess [2]. Widening of these soft tissues is pathologic until proven otherwise as seen in our index case (Figure 1). The measurement of the distance from the anterior surface of the C2 vertebra to the posterior border of the airway should be 7 mm or less, regardless of the patient's age [4]. With measurement starting at the C6 vertebra, this width should be 14 mm or less in children younger than 15 years of age and 22 mm in adults. A simpler but less precise rule is that on soft tissue plain X-rays, the prevertebral body should be less than one half the width of the corresponding vertebral body. However, in our index case, it was about three times the size of the vertebral body, which is an unusual presentation (Figure 1) [3-5]. Some authors have reported the use of computed tomographic scans to diagnose retropharyngeal abscess, especially in uncommon situations [5], the authors have no knowledge of such report in our region.

Prompt surgical intervention with drainage of the abscess was the most essential part of the management of this patient, especially in view of the size of the obstruction and the gas content, as the possibility of rupture was envisaged because of the challenges of our ICU, an inadequate laboratory facility and insufficient personnel. We consider prompt surgical intervention to have been a lifesaving step in the present case. The index case was intubated despite some difficulty because of the enlarged retropharyngeal mass, deviated trachea (Figure 2) and narrowed pharyngolaryngeal space under direct visualization. Previous reports have proposed fiberoptic intubation, which was not available in our center, or cricothyroidotomy or, in the worst case scenario, tracheostomy [4], all of which are done to protect the lower airway. Positioning the airway correctly and avoiding unnecessary manipulation is essential [3,4,9]. The patient is at risk of compression the pharynx or trachea with possible suffocation or rupture with asphyxiation or aspiration of the abscess, sepsis and pneumonia if left unattended to or at intubation in the hand of inexperienced anesthetist, as seen in the X-ray of this patient. Some workers have reported the relapse of retropharyngeal abscess despite drainage [5], and other complications are highlighted above. The specimen obtained in our present case was transported to our sister medical center where it was cultured and reported.

Delays in diagnosis and treatment can lead to the risk of complications. The mortality of retropharyngeal abscess is due to the association with airway obstruction, mediastinitis, aspiration pneumonia, epidural abscess, jugular venous thrombosis, carotid artery erosion, pericarditis and airway compromise.

Our patient was extubated but still developed laryngeal spasm, which is an uncommon situation that requires close monitoring immediately after surgery. Laryngeal spasm in a standard setup is an indication for ICU admission, which is lacking in our center; however, our patient was managed in the recovery room by manual ventilation and monitoring of vital signs. Laryngospasm is a forceful, involuntary spasm of the laryngeal musculature caused by stimulation of the superior laryngeal nerve, which is the sensory innervation of the larynx. Its signs include an inability to ventilate the patient with rapid desaturation. Prevention can be achieved by extubating the patient using a no-touch technique when the patient is awake [14], as was done in the index case, or under deep anesthesia (possibly after a magnesium infusion) [15], which was not available in our center in the event that the awake extubation failed. Complications of laryngospasm can be prevented through application of a gentle jaw thrust, but if this fails, the depth of anesthesia can be increased with intermittent positive pressure ventilation on a ventilator, which is not available in our center. Some researchers have used propofol to increase the depth of anesthesia because of its rapidity of onset and predictability [16]. However, in the index case, halothane was used.

## Conclusion

This case report highlights an unusual presentation and management of retropharyngeal abscess. The presence of gas-forming organisms in this clinical scenario makes it an interesting case. Physicians should maintain a high index of suspicion, however, when encountering children with torticollis or unexplained neck pain or swelling and should perform the necessary investigations to avoid delay in diagnosis, which might lead to serious consequences. There also need to be close monitoring of the patients immediately after surgery and readiness for challenges even in the face of inadequate facilities. Despite numerous challenges encountered during the management of our patient, the end result was satisfactory. This report is expected to affect positively clinical practice in the field of ENT surgery, anesthesia and medicine in general in resource-challenged settings such as ours.

## Consent

Written informed consent was obtained from the patient's parents for publication of this case report and accompanying images. A copy of the written consent is available for review by the Editor-in-Chief of this journal.

## Competing interests

The authors declare that they have no competing interests.

## Authors' contributions

AOA was the principal surgeon, performed the literature search and prepared the manuscript and takes responsibility for the publication. FJO assisted in preparing and proofreading the manuscript for intellectual content and gave final approval for the publication. OEO was the anesthetist, obtained the accompanying images and conceived the idea for the manuscript. OSA did the literature search, contributed to the preparation of the manuscript and reviewed the manuscript. All authors read and approved the final manuscript.
